# Afoxolaner as a Treatment for a Novel *Sarcoptes scabiei* Infestation in a Juvenile Potbelly Pig

**DOI:** 10.3389/fvets.2020.00473

**Published:** 2020-09-08

**Authors:** Joe S. Smith, Darren J. Berger, Sarah E. Hoff, Jeba R. J. Jesudoss Chelladurai, Katy A. Martin, Matthew T. Brewer

**Affiliations:** ^1^Food Animal and Camelid Hospital, Veterinary Diagnostic and Production Animal Medicine, College of Veterinary Medicine, Iowa State University, Ames, IA, United States; ^2^Systems Modelling and Reverse Translational Pharmacology, College of Veterinary Medicine, Iowa State University, Ames, IA, United States; ^3^Veterinary Clinical Sciences, College of Veterinary Medicine, Iowa State University, Ames, IA, United States; ^4^Department of Veterinary Pathology, College of Veterinary Medicine, Iowa State University, Ames, IA, United States

**Keywords:** afoxolaner, isoxazoline, pig, *Sarcoptes scabiei*, zoonosis

## Abstract

A 2 months old female Vietnamese potbellied pig presented to a veterinary teaching hospital with a referring complaint of pruritus. A human caretaker of the pig had recently been diagnosed with a *Sarcoptes* spp. dermatitis. Microscopic examination of the skin scrape samples and BLAST analysis confirmed the species of the mite as most closely related to *Sarcoptes scabiei* var. canis (AY493391). The pig was treated with afoxolaner as previous treatment with ivermectin was not efficacious. Recheck examinations and follow up revealed the pig to be non-pruritic and resolving. Afoxolaner may be a therapeutic option when treating *Sarcoptes* spp. infections in companion pigs.

## Introduction

Isoxazolines are a veterinary drug class that have recently become available for the prevention of ectoparasites of cats and dogs. Available via oral and dermal application, with both forms leading to systemic distribution, they maintain long periods of activity after a single dose (1). These drugs bind gamma-aminobutyric acid gated chloride receptors as well as l-glutamate chloride channels. This receptor binding then results in the death of the parasite (2). The isoxazolines selectively target arthropod receptors over mammalian receptors and as such have been used for both experimental and naturally occurring flea, tick, and mite infections in dogs and cats (2–5).

Sarcoptic mange is an occasional infection in commercial pigs, and outside of a few case reports is not well-described in companion miniature pigs, such as Vietnamese potbellied pigs. When sarcoptic mange is encountered in pigs, risks include zoonotic transmission to humans in close contact (6). Previously reported treatments comprise injectable ivermectin combined with chlorhexidine shampoo (6). Currently no case reports describe the use of an isoxazoline drug, such as afoxolaner for the treatment of sarcoptic mange in a companion miniature pig.

Companion miniature pigs are increasing in popularity as pets in North America, with some estimates suggesting the total population being in the hundreds of thousands (7). Although all swine are considered food animals in the United States, many owners of potbellied pigs consider them companion animals. This presents a challenge to veterinarians, when making treatment decisions involving extralabel drug use. With their demonstrated efficacy in small animals, the isoxazoline class of drugs presents a potential option for the treatment of external parasites in miniature companion pigs. In this report, we demonstrate the usefulness of a commercial formulation of afoxolaner for the treatment of *Sarcoptes scabiei* var. canis in a juvenile potbelly pig.

### Case Presentation and History

A 2-months-old, 4.3 kg, intact female Vietnamese potbellied pig presented to Iowa State University Food Animal and Camelid Hospital to evaluate pruritus of 2 weeks duration. The owner had noticed pruritus and alopecia, and had administered one dose of ivermectin (0.6 mg/kg, PO). The client noticed no change in clinical condition following treatment. Shortly after the pig was noted to be pruritic, a resident of the home, who had close contact with the pig, developed skin lesions and pruritus, which was diagnosed by the resident's primary care physician as suspect *scabies*. The pig was examined by a board-certified-large animal internal medicine specialist as well as a board-certified veterinary dermatologist. Parasites obtained from skin scrapings were evaluated by a board-certified parasitologist, and additional material was retained for molecular characterization.

## Materials and Methods

### Molecular Characterization

For genetic confirmation of mite identity, a portion of the mitochondrial cytochrome C oxidase subunit 1 (Cox 1) was amplified (8). DNA was extracted from mites present in the skin scraping using the DNeasy blood and tissue kit (Qiagen, Valencia, CA), using the manufacturer's protocol. Mt-Cox1 gene was amplified in 50 μL reactions with 2 μL of extracted DNA, 3 mM of MgCl_2_, 200 μM of dNTPs, 1x PCR buffer, and 2 units of Taq DNA polymerase (GoTaq Flexi, Promega, Madison, WI), and 2.5 μM of primers ScabCox1F 5'- GACACCCAGAAGTTTACATTC-3' and ScabCox1R 5'- TATATTTTGATAATGAATCTC-3' (9). PCR conditions were 94°C for 2 min, 30 cycles of 94°C for 30 s, 45°C for 15 s, 72°C for 1 min, and 72°C for 5 min. PCR amplification was confirmed by gel electrophoresis in 0.8% agarose gels. Amplification products were purified using the PureLink PCR purification kit (ThermoFisher, Waltham, MA, USA), and sequenced on an Applied Biosystems 3730 × l DNA analyzer. Contigs were assembled using CAP3 (10). Nucleotide BLAST analysis was carried out on GenBank, and 34 mt-Cox I gene sequences of *S. scabiei* and one of *Amblyomma americanum* were retrieved from the GenBank nucleotide database. Multiple sequence alignments were created using Clustal omega (11). The best phylogenetic model was selected using SMS (12) and maximum likelihood phylogenetic analysis using the Hasegawa-Kishino-Yano model with invariable sites (HKY85+I) was conducted in Mega X (13). Haplotype analysis was carried out using DnaSP 6 (14). One hundred and two total S. scabiei cox I sequences obtained from GenBank and the present study were analyzed at 385 sites. Fourty-four unique haplotypes were identified. The cox I sequence from the present study belongs to a novel haplotype.

## Results

### Physical Examination

**Figure 1:**
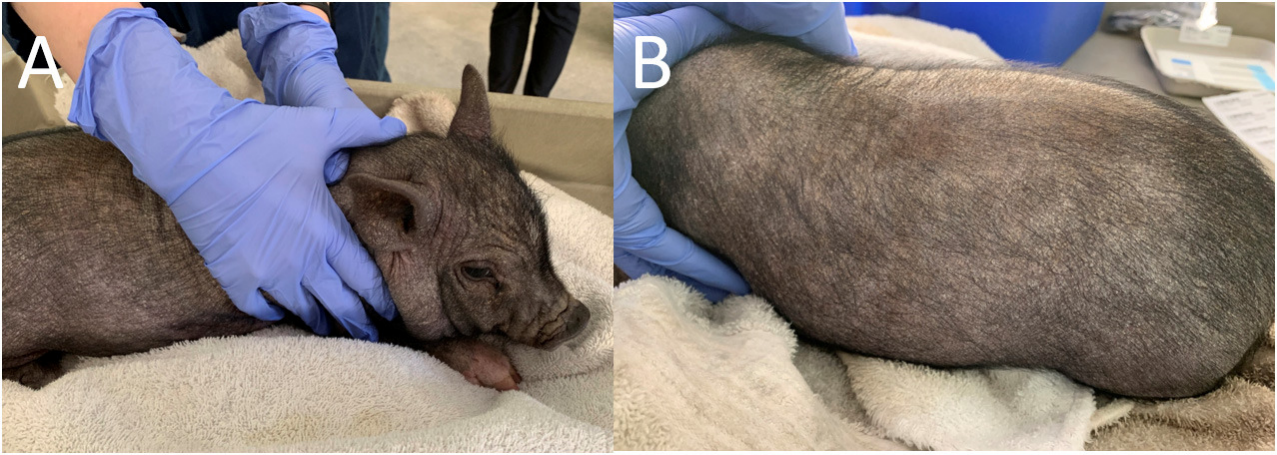
Photographs of the pig taken on initial examination. Note the bilateral excoriations. **(A)** Right Lateral View. **(B)** Left Lateral View.

Dermatological examination on the initial visit revealed the patient had moderate diffuse generalized erythema and with generalized hypotrichosis. Mild diffuse generalized crusting with scattered papules along the dorsal and ventral trunk were also present. More focally moderate crusting was appreciated along the pinnal margins and the cranial dorsum. Moderate to marked diffuse scale was more prominent along the dorsal surfaces. Mild excoriations were present over the right and left lateral thorax and caudal aspects of the forelimbs, bilaterally (Figure 1). Minimal manipulation of the patient's dorsum or head was noted to elicit severe pruritic activity. The remainder of physical examination findings were within normal limits.

### Parasite Morphology

**Figure 2:**
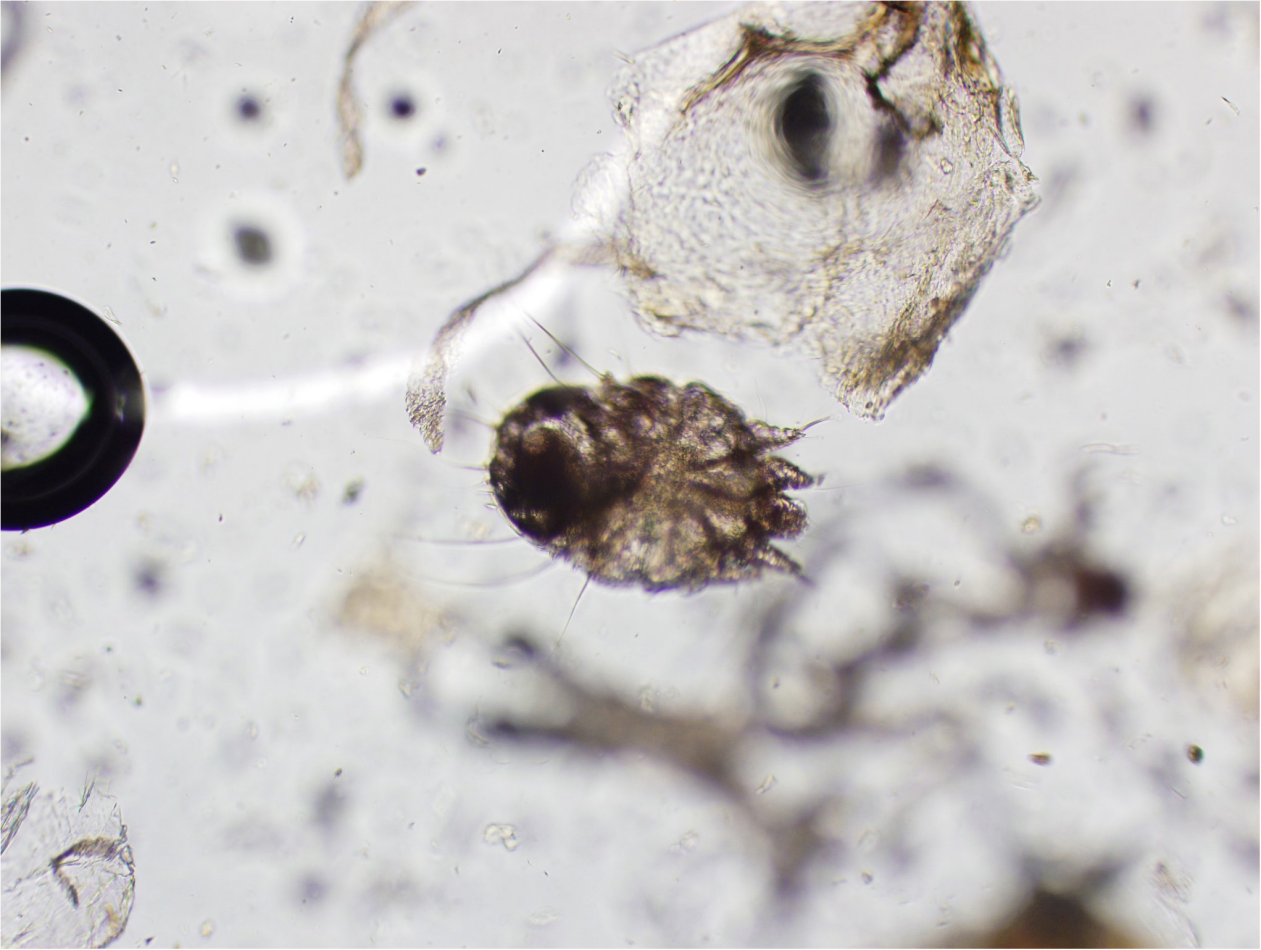
Mite identified from skin scraping (magnification: 20X). Note the long unjointed pedicles, cuticular spines, setae, and terminal anus consistent with Sarcoptes spp.

Skin scrapings revealed sarcoptiform astigmatid mites bearing long unjointed pedicles, cuticular spines, setae, and a terminal anus (Figure 2). Taken together, the morphological features of the mites were consistent with *Sarcoptes* spp.

### Molecular Identification

**Figure 3:**
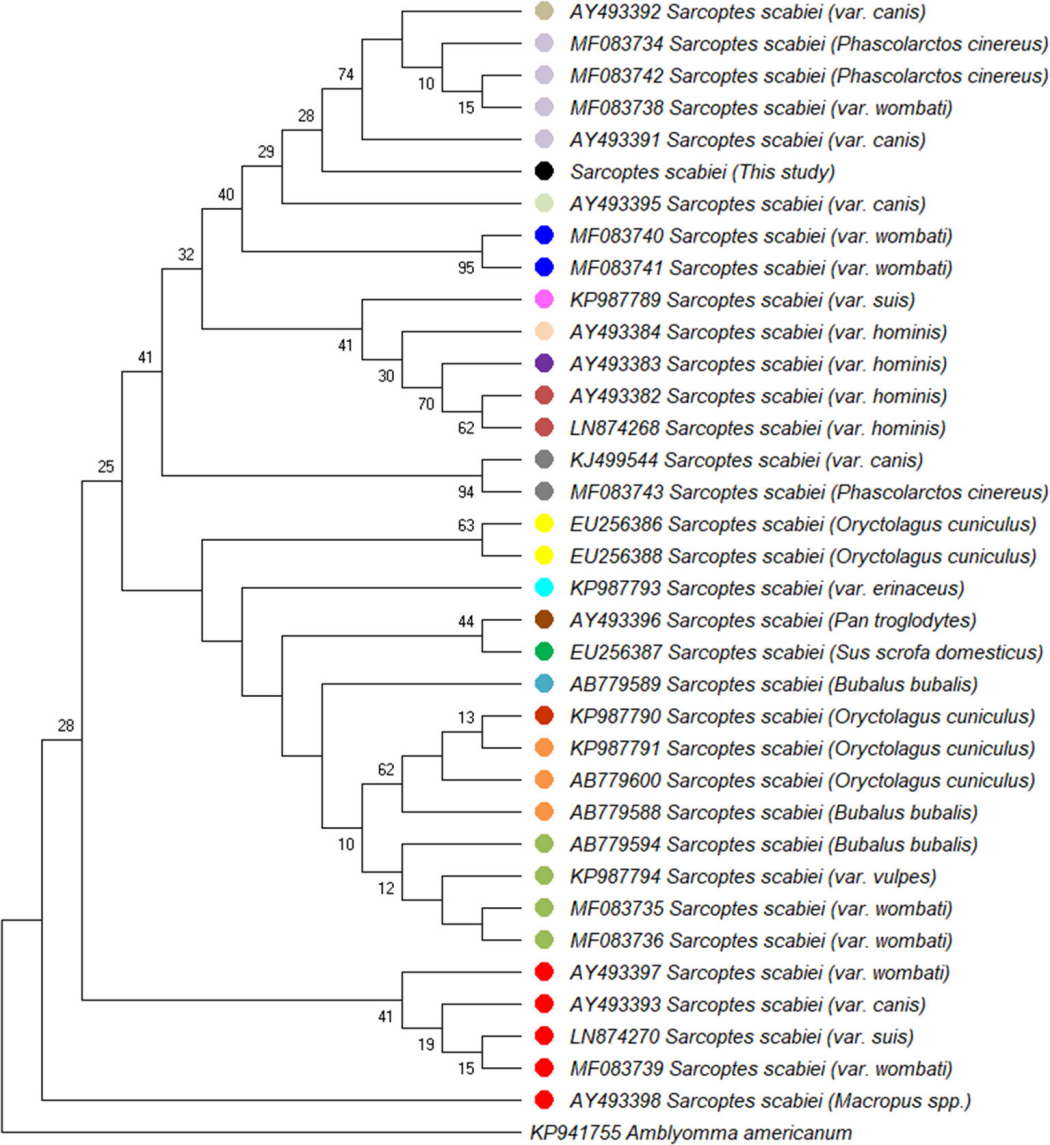
Maximum likelihood phylogenetic analysis of 36 partial mt-cox1 sequences analyzing 682 basepairs in each sequence using the HKY85+I method. The percentage of trees in which the associated taxa clustered together is shown next to the branches. The sequence from the present study is indicated (·). Sequences obtained from GenBank records are indicated by accession numbers.

A partial amplicon of the mitochondrial cytochrome c oxidase subunit I gene was amplified by PCR from mite DNA and sequenced (GenBank: MN985652.1). Nucleotide BLAST analysis of the 722 bp product revealed that the pig isolate in the present study had the highest identity with *S. scabiei* var. canis (AY493391). BLAST results are listed in Table 1. Phylogenetic analysis showed that the pig isolate was in the same cluster as sequences from *Sarcoptes scabiei* isolated from dogs, koalas, and wombats. Figure 3 represents a maximum likelihood analysis of the genotype from this study.

**Table 1:** Nucleotide BLAST results of the partial mitochondrial cytochrome c oxidase subunit I product from this study.

**GenBank**	**Parasite**	**Variant/Host**	**Partial mt-Cox**
**Accession**			**I sequence**
**number**			**identity**
AY493391	*Sarcoptes scabiei*	var. canis	99.72%
MF083742	*Sarcoptes scabiei*	Host: Koala *Phascolarctos cinereus*	99.58%
MF083738	*Sarcoptes scabiei*	var. wombati	99.58%
MF083734	*Sarcoptes scabiei*	Host: Koala *Phascolarctos cinereus*	99.58%
LN874268	*Sarcoptes scabiei*	var. hominis	99.58%
AY493395	*Sarcoptes scabiei*	var. canis	99.58%
AY493382	*Sarcoptes scabiei*	var. hominis	99.58%
AY493392	*Sarcoptes scabiei*	var. canis	99.45%
AY493383	*Sarcoptes scabiei*	var. hominis	99.45%
MF083741	*Sarcoptes scabiei*	var. wombati	99.31%
MF083740	*Sarcoptes scabiei*	var. wombati	99.31%
MF083736	*Sarcoptes scabiei*	var. wombati	99.31%
MF083735	*Sarcoptes scabiei*	var. wombati	99.31%
AY493396	*Sarcoptes scabiei*	Host: Chimpanzee *Pan troglodytes*	99.31%
MF083739	*Sarcoptes scabiei*	var. wombati	99.17%
LN874270	*Sarcoptes scabiei*	var. suis	99.17%

### Treatment

Due to the treatment failure of the previously administered ivermectin, an ~2.6 mg/kg dose of afoxolaner was administered orally, with instructions to repeat in 1 month. The pig was closely monitored for 72 h after administration and no adverse effects were noted. The clients were instructed to thoroughly clean the pig's bedding and limit contact with other pigs and people in the household until after resolution of pruritus.

### Follow-Up Examination

**Figure 4:**
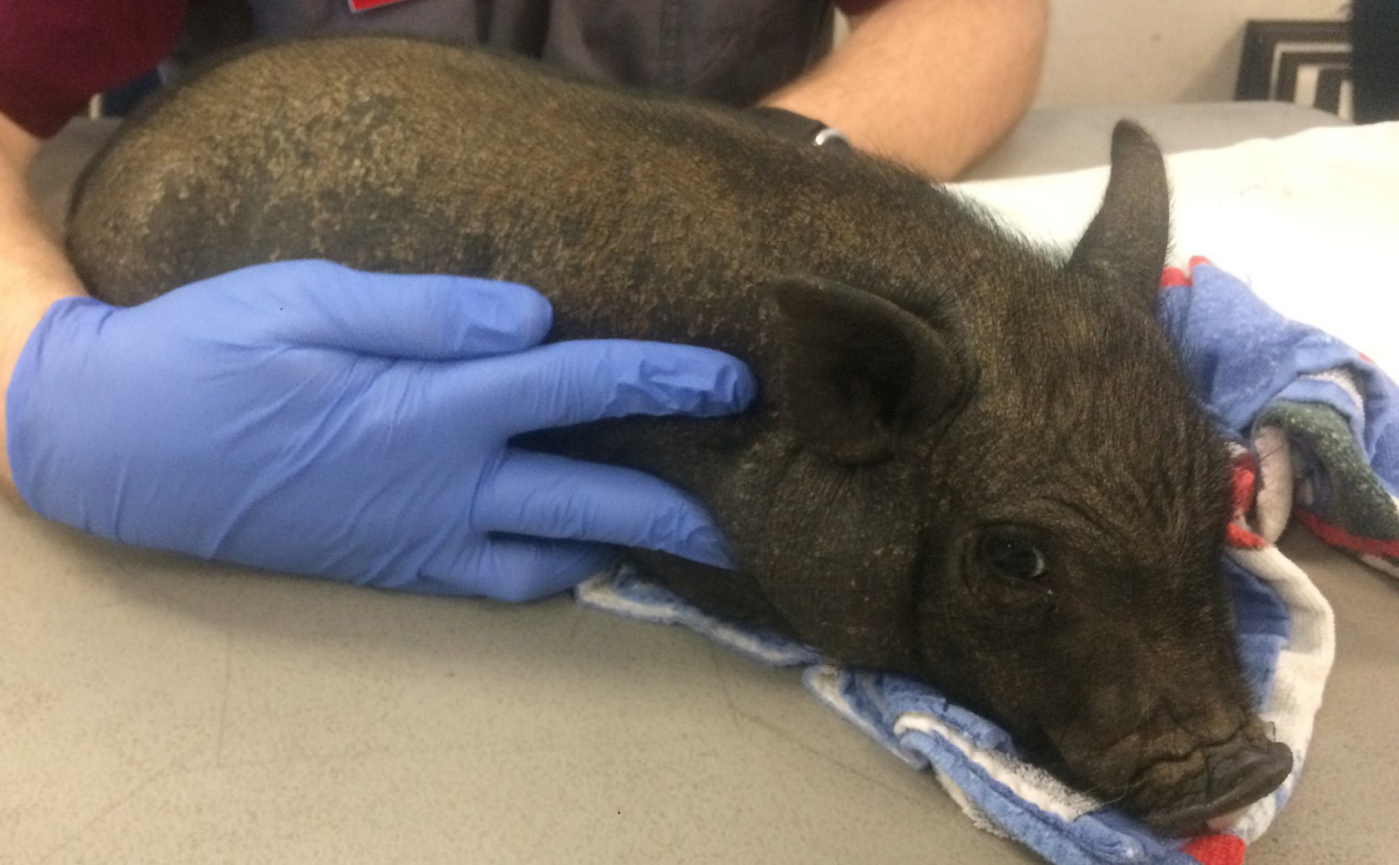
Photographs from recheck examination. Note the hair regrowth, increased dorsal scaling and resolution of the previously examined excoriations.

Three weeks after the initial examination the pig was re-evaluated. Physical examination findings were within normal limits and a dermatologic examination revealed widespread crusting along the dorsum, no active pruritis or superficial inflammation was noted (Figure 4). The owner reported that pruritic behavior had ceased ~4 days after administration of afoxolaner. Microscopic examination of the skin revealed no live mites, or eggs. Follow up communication 8 and 16 weeks after examination revealed resolved alopecic areas and no pruritus observed since the original episode.

## Discussion

Sarcoptes infections cause sarcoptic mange in domestic and wild mammals. The genus *Sarcoptes* is thought to contain a single species, *S. scabiei*, with host-adapted variants. In pigs, sarcoptic mange can occur as an acute cause of pruritis in fattening pigs, and a chronic herd problem in adults, which can be difficult to eliminate from a herd (15). Because multiple treatments are often required to clear infections, sarcoptic mange cases can appear clinically resistant to avermectins (16, 17). While not commonly reported in miniature companion pigs, zoonotic transmission is possible (6). There are significant gaps in understanding host specificity in *Sarcoptes scabiei*. It is of note that in the present study, mites isolated from pigs were closely related to isolates from dogs and Australian marsupials. There have been reports of *S. suis* infections (9), but not *S. canis* in pigs as of this case report. While injectable ivermectin, dosed at 0.3 mg/kg for two subcutaneous injections (q 14 d) has been effective when combined with chlorhexidine baths in potbellied pigs (6), a relative paucity of information exists for the efficacy of this drug when administered orally to miniature pigs. In this case, the patient presented to a university hospital with an infection suspected to be resistant due to past treatment failure.

In production pigs oral ivermectin administration has been demonstrated to aid in controlling mange (18). At this time it is unknown why the previous oral administration of ivermectin was not clinically efficacious in this patient, and as the bottle was not located it is possible that this product was expired or otherwise not administered appropriately by the client. As the drug administration was not witnessed, it is possible that the pig expelled the ivermectin shortly after administration. Resistance to therapy, as postulated as emerging in human scabies infections (19), could also be a potential reason for treatment failure in this case. However, this cannot be absolutely confirmed by this case, as the only observation was a lack of efficacy.

Afoxolaner is an isoxazoline that blocks native and expressed arthropod GABA-gated chloride channels, including wild-type and channels possessing the resistance to dieldrin (A302S) mutation (20). In dogs and cats it has demonstrated considerable efficacy for ectoparasite control for fleas (2, 21), ticks (21), and mites including *Sarcoptes* (5), *Otodectes* (3), and *Demodex* (22, 23). With the selectivity for invertebrate channels the isoxazolines are thought have a wide margin of safety in mammals. While commonly used in dogs with rarely reported adverse effects, there have been reports of neurological toxicity (i.e., seizures) associated with this class of antiparasitics (24). Our case, and a research project using isoxazolines in pigs as a model for human disease (25) observed no neurological effects, suggesting that these drugs may be safe for use in pigs.

Cox I amplified from the porcine isolate in the present study had the highest identity with *S. scabiei* var. canis isolated from the USA, but was not 100% identical. While microsatellite genotyping demonstrates geographical separation between isolates and biological adaptation to the herbivore-, omnivore-, and carnivore- host groups (26), Cox I haplotype variation does not completely correlate with established host variants. Additionally, association between host and named variants are not absolute due to cross host species transfer due to host interactions (27). Further research is needed to determine the affinity of *S. scabiei* var. canis for porcine infestations.

At this time the authors were unable to identify other naturally-occurring cases of *S. scabiei* var. canis infections in pigs. Experimental transmission has been documented previously (28), however that study utilized microscopy, instead of sequencing for mite identification. Traditionally, causes of sarcoptic mange in pigs are described as caused by *S. scabiei* var. suis (15, 18). The mitochondrial subunit 1 cytochrome c oxidase (COX-1) marker is an informative genetic marker for characterization of *S. scabiei* (29). Analysis of COX-1 markers from our case identified a 99.72% sequence identity with *S. scabiei* var. canis AY493391. It has been suggested that the host preference is partially correlated with genetic differences within *S. scabiei* sequences (9), however it is uncertain at this time where the source of infestation was for our case. Regardless, *S. scabiei* var. canis infestation has been described in immunocompromised human patients (30), so clinicians and clients should be aware of the zoonotic implications of infestations of the mite in pigs.

Companion miniature pigs would be considered a food animal in the United States from a regulatory perspective (7, 31). Although uncommon, there are instances where companion pigs have entered the food chain (32). As such, clinicians should be aware of the regulatory requirements involving extra-label drug use in these species. The use of afoxolaner in this case was justified by the veterinary care team as treatment failure with ivermectin was suspected, and there was a need for a rapid resolution due to the human case observed by the owner. Additionally, while ivermectin is labeled for the treatment of *S. scabiei* var. suis infestations in pigs, there are regional differences in available formulations, and afoxolaner has demonstrated efficacy for treatment of this mite in naturally infested dogs (5). While no withdraw information exists for afoxolaner, a similar compound, fluralaner is currently labeled in Europe for use in poultry for the treatment of red mite (*Dermanyssus gallinae*) with a 14 days meat withdrawal and 0 day egg withdrawal period.

This report details the use of afoxolaner for the treatment of Sarcoptes in a companion miniature pig presented to a veterinary teaching hospital for pruritus caused by suspected infestation with *Sarcoptes scabiei* var. canis. Due to prior unsuccessful treatment with ivermectin the pig was administered afoxolaner and had resolution of pruritus within 96 h of administration. No adverse effects were noted in the patient after administration of the drug. Afoxolaner and other isoxazolines may prove to be effective therapies for miniature companion pigs with ectoparasites.

## Data Availability Statement

The datasets presented in this study can be found in online repositories. The names of the repository/repositories and accession number(s) can be found below: https://www.ncbi.nlm.nih.gov/genbank/, MN985652.

## Ethics Statement

Written informed consent was obtained from the owner for the participation of their animal in this study.

## Author Contributions

JS, DB, and SH diagnosed and managed the clinical case. JJ, KM, and MB performed BLAST analysis, as well as consulted on the clinical case. All authors contributed to manuscript construction.

## Conflict of Interest

The authors declare that the research was conducted in the absence of any commercial or financial relationships that could be construed as a potential conflict of interest.
